# Transition to Kindergarten for Children on the Autism Spectrum: Perspectives of Korean–American Parents

**DOI:** 10.1007/s10803-022-05665-1

**Published:** 2022-07-12

**Authors:** Sohyun An Kim

**Affiliations:** 1 University of California, Los Angeles, USA; 2 California State University, Los Angeles, USA

**Keywords:** Transition to kindergarten, Autism spectrum disorder, Parent perspectives, Korean–American parents, School readiness

## Abstract

This study explores Korean–American parents’ perceptions on successful transition to kindergarten (TTK) for their child on the autism spectrum. It further examines challenges experienced during this process, and possible predictors for their challenges. Findings from an online survey (N = 212) indicate that participants consider their child’s behavioral readiness and cooperation with teachers as the most important school readiness skills for successful TTK. They further consider building positive relationships with teachers and providing support at home as the most important support parents could provide during this process. Moreover, the child being a vocal communicator, higher income and parent’s educational level were found to buffer against their reported challenges, while first-generation immigrant status and restrictive school placement were found to predict more challenges.

Research has consistently emphasized the importance of transition to kindergarten in fostering future school success (Cooper et al., 2010; Daley et al., 2011; Eckert et al., 2008; Forest et al., 2004; Gill et al., 2006; McIntyre et al., 2006, 2007, 2010; Starr et al., 2016). However, transition to kindergarten (TTK) can be a stressful milestone for the child as well as for the entire family (DeCaro & Worthman, 2011; McIntyre et al., 2010; Welchons & McIntyre, 2015, 2017) and it requires the parents and educators to work together to ensure success. Therefore, it is important to examine the perspectives of parents when their child undergoes such a critical milestone in their academic journey. It would be especially beneficial to investigate child-level factors, family-level factors, and environmental factors that may contribute to the experiences of parents who are at a complex intersectionality of cultural and linguistic diversity and neurodiversity.

## Transition to Kindergarten for Children on the Autism Spectrum

The Office of Head Start (OHS) defines school readiness as possessing the skills, knowledge, and attitude for school success, which include physical, social, and cognitive development (Head Start, 2018). Similarly, prior studies indicate that entering kindergarten students are expected to communicate with teachers and peers using socially acceptable school-based conventions, to sit and attend to classroom instructions for extended periods of time, to cooperate with teachers, to engage in cooperative play with peers, and to recognize others’ feelings (Eckert et al., 2008; Gill et al., 2006; LoCasale-Crouch et al., 2008; Puccioni, 2018; Welchons & McIntyre, 2017).

Accordingly, the TTK process may pose unique challenges to students with developmental disabilities such as autism spectrum disorder (ASD) when their support needs and readiness skills differ from their neurotypical peers. Extant studies indicate that parents of children with atypical development report significantly more concerns surrounding their child’s overall readiness for kindergarten than parents of typically-developing children (McIntyre et al., 2010; Welchons & McIntyre, 2015). Similarly, teachers perceive students with disabilities to have more kindergarten entry challenges such as externalizing behaviors and poorer overall student–teacher relationships, when compared to children without disabilities (McIntyre et al., 2006; Welchons & McIntyre, 2017). Such findings highlight the heightened vulnerability of children on the autism spectrum in the transition process. However, little is known about specific child-level factors that may contribute to the overall challenges children on the autism spectrum and their parents experience during their TTK.

## Authentic Relationship Between Families and Educators

TTK is not only a process for children; it requires the whole family to make adjustments. However, this adjustment can be especially taxing for families with children with disabilities (DeCaro & Worthman, 2011; McIntyre et al., 2010; Welchons & McIntyre, 2015, 2017) when their priorities in support needs do not align with the priorities of educators. Therefore, supporting the transition to school requires the building of trusting relationships between families and educators (Gill et al., 2006; Starr et al., 2016).

Family engagement in the context of early childhood system refers to the systematic inclusion of families in activities and programs that promote children’s development, learning, and wellness, including in the planning, development, and evaluation of such activities, programs and systems (U.S. Department of Health & Human Services, 2016). Research supports that engaging families as essential partners in children’s learning and development positively impacts their long-term outcomes (U.S. Department of Health & Human Services, 2016). More specifically, recent studies affirm the importance of the family-educator partnership in ensuring effective disability-related services (Balcells-Balcells et al., 2019; Davis & Gavidia-Payne, 2009; Summers et al., 2007). For example, having a positive family-educator relationship contributed to parents experiencing desired practices at the school. However, about half of the parents with children with disabilities who desired family-centered practices reported as not experiencing such practices during their early elementary years. (Dunst & Hamby, 2019). Considering that over 90% of the participants in this study identified themselves as White, challenges faced by parents from culturally and linguistically diverse (CLD) backgrounds may be more exacerbated than they appear in the study. However, there is a paucity of research examining experiences of parents from a CLD background and how their family-level factors may impact their TTK process.

The American school system is becoming increasingly diverse, and such diversity may create a mismatch between families and educators when they do not share the same language, culture, and knowledge of the school system. For instance, parents from “non-majority” cultural backgrounds may face additional challenges and stressors when navigating the school system due to the linguistic barriers, socioeconomic status and cultural values and belief system that misalign with the educators’. Further, while parents of children on the autism spectrum from a CLD background acknowledge relationship building with the educators to be an essential component of a successful TTK, they perceive this to be challenging when there is a cultural mismatch between parents and educators in the handling of the relationship, in ways they advocate for their children, and their communication styles (Starr et al., 2016). Such misalignments may serve as additional barriers for Korean–American parents in building authentic relationship with their child’s educators and navigating the school system when their child transitions to kindergarten.

## Parent Involvement and Cultural Misalignment

Educators underscore the importance of parent involvement in building the necessary school-readiness skills, and they consider that successful TTK depends upon parents playing a proactive role in preparing their child for the transition, actively seeking information, participating in school-related activities, and advocating for their child’s needs (Gill et al., 2006; Puccioni, 2018).

However, CLD parents are often viewed by educators as less involved in their children’s school, and such a perspective may decrease the teachers’ invitation for involvement, which in turn, discourages parental involvement in the transition process for these parents (Kim, 2009). This may lead to the misconception that these parents lack interest in their children’s education and that they are less invested in their participation at home (Kim, 2009). Moreover, cultural differences in the parenting role between mother and father, communication styles and approaches in supporting their children may create additional barriers to building meaningful and trusting relationships between families and educators (Cooper et al., 2010; Starr et al., 2016).

More specifically, parents from collectivist cultures such as Korea find it disrespectful to voice their opinions or initiate communication with educators to discuss their concerns. Stemming from their belief in Confucianism, Korean parents do not tend to regard themselves as equal partners with teachers in educating their children, but rather consider their role as exclusively in the home (Sohn & Wang, 2006). Such beliefs may create an additional barrier for Korean–American parents to building a meaningful and effective relationship with the educators.

Further, prior studies indicate that Korean mothers are typically very invested in their children’s education and regard the American school system highly (Kim, 2009; Sohn & Wang, 2006). However, they are hesitant to take an active role at school (e.g., PTA President) or voice their opinions because of a much higher respect they have towards school and therefore viewing the school as an authoritative entity. Moreover, Korean parents tend to be reserved and indirect when concerns arise or when they have suggestions, and they are likely to delegate their children’s education to their teachers. Such indirect interactions are considered a way to show their respect for teachers’ authority (Jung, 2011; Sohn & Wang, 2006). Such attitudes may be perceived by educators in the U.S. as being indifferent about their child’s education or being unwilling to get involved.

Comparing these perspectives, there is a disparity between educators’ expectations and the Korean–American parents’ attitude towards the school system. In other words, the mismatch between Korean–American parents and educators’ cultural perspectives toward the school system and their communication style may function as a barrier in building a collaborative relationship between the two stakeholders.

Despite these unique cultural perspectives of Korean–American families towards the U.S. school system and ASD, and the disparities between the viewpoints of parents and educators, little is known about the TTK experiences for Korean–American parents with a child on the autism spectrum and environmental factors that may contribute to their unique challenges when navigating the school system. Although there has been an increase in the research base in Asian cultural beliefs in developmental disabilities and the relationship with the school system, treating the Asian population, which includes many distinct nationalities and cultural backgrounds, as a homogeneous group may bring forth an inaccurate representation (Else-Quest & Hyde, 2016). Rather, a closer examination of unique variables of subgroups and considerations for intersectionality for a given population is necessary in order to avoid inadvertently remarginalizing certain subgroups.

Furthermore, according to the U.S. Census Bureau (2010), the Asian population has been the fastest-growing population in the US. between 2000 and 2010, while Koreans account for 9.9% of the total Asian population in the US. Further, Asian American children are reported to be overrepresented in the category of ASD in special education (Marks & Kurth, 2013).

In spite of the continued increase in this population, there is a paucity of research related to the TTK of children on the autism spectrum in general, and far less is known about experiences of Korean–American families. Therefore, the current study aims to contribute to this gap in the literature by examining the unique challenges and barriers faced by Korean–American families with children on the autism spectrum and to evaluate how they perceive their experiences with the transition process.

## Current Study

The purpose of this quantitative and exploratory study is to examine Korean–American parents’ experiences and perspectives when their child on the autism spectrum transitions to kindergarten. It further aims to examine how parents’ linguistic factors and cultural beliefs regarding the U.S. school system and their child’s ASD may influence the challenges they experience. The following questions are addressed in this study using survey methodology:

1. How do Korean–American parents define a successful transition to kindergarten for their child on the autism spectrum?
2. What are the challenges and barriers Korean–American parents report when their child on the autism spectrum transitions to kindergarten?
3. What are the protective and risk factors for challenges reported by Korean–American parents when their child on the autism spectrum transitions to kindergarten?

## Method

### Participants

The sample included 212 Korean–American parents (59 self-reported as 1st generation immigrant, 24 reported as 1.5 generation immigrant, and 129 self-reported as 2nd generation immigrant parents) who had a child or children on the autism spectrum who transitioned to kindergarten within the past 6 years. For this study, the following definitions were used for immigrant generations: first generation Korean–American immigrant is defined as those who immigrated from South Korea to the United States as an adult and who completed elementary and secondary education (K-12) in Korea. 1.5 generation Korean–American immigrant is defined as those who immigrated from South Korea to the U.S. as a minor and who completed some or all of K-12 education in the U.S. Second generation Korean–American immigrant is defined as those who have a Korean heritage background who were born in the U.S. Second generation immigrants also completed K-12 education in the U.S. These definitions were specified verbatim on the survey form and participants were asked to choose the one that best described their immigration status.

The sample size was determined by conducting a power analysis using G*Power 3 software (Faul et al., 2009) using the data from a pilot study conducted with 62 participants (Kim, 2019) addressing the same research questions. Results from the power analysis suggested that 200 participants would comprise an adequate sample size for this study.

Inclusion criteria for participating in the study were (1) Korean–American parents residing in the U.S., (2) respondent self-reported as having a child diagnosed with ASD, and (3) child with ASD transitioned to kindergarten within the last 6 years. All participants met the inclusion criteria based on their self-reported responses.

Participants were recruited via online platforms that are specifically targeted for Korean–American parents or Korean–American parents with a child with ASD. Examples include a closed Facebook group for Korean–American parents and a membership-based online discussion forum for parents with a child with ASD within a portal site for Korean–American mothers. In addition, a recruitment flyer was emailed to a community-based Korean–American organization called Korean Community Center, a non-profit disability service organization called Easterseals Southern California, and a Regional Center in Los Angeles, California. Personnel in these organizations shared the flyers with parents who may have a child with ASD. These recruitment modalities were carefully selected as a way to account for the anonymous nature of the survey and for the self-reported inclusion criteria (e.g., diagnosis of ASD).

### Procedures

All procedures were approved by the Institutional Review Board of the University of California, Los Angeles (IRB # 20-00436). The survey instrument was created on Qualtrics, and it was disseminated via anonymous online link. The recruitment flyer, informed consent, and the survey were available in both Korean and English. They were first written in English, then translated by a professional translator. The translated version was reviewed and edited by the author, who is bilingual in English and Korean, to ensure accuracy.

Upon receiving the survey link, interested parents had an opportunity to review the information about the study and the informed consent document. The first three questions of the survey were used for validation purposes. The first question asked for the respondents’ consent to participate. The second question asked if the participant considered themselves as 1st, 1.5 or 2nd generation Korean–American immigrants, or if they do not consider themselves as Korean–American. If they chose the last choice, the survey was discontinued per the exclusion criteria. The third question asked if they had a child on the autism spectrum who transitioned to kindergarten within the last 6 years. If the answer was no, the survey was discontinued. At the end of the survey, participants had the option to enter their email address to receive a $10 Amazon e-gift card. Their email addresses were used for this purpose only, and they were deleted from the database afterwards. The median length of time for survey completion was approximately 15 min.

### Measures

The survey instrument was adapted from The Family Experiences and Involvement in Transition (McIntyre et al., 2007) and Elements for Transition to Kindergarten (Forest et al., 2004). The adaptation process included adding culturally-relevant survey items such as cultural values, stigmas, linguistic barriers, and discrimination. Additionally, common characteristics of ASD (e.g., challenges in social communication, self-care skills, behavioral readiness, sensory needs, etc.) were taken into consideration when creating the survey.

The initial prototype was field-tested by eight professional and community members, six of whom were Korean–American bilingual mothers. Average duration was measured, and feedback regarding clarity of the survey questions was sought. Then the survey instrument was revised based on their feedback. Feedback regarding wording, content validity and relevance for the Korean–American population was also provided by the professional translator after the revision process. This version of the survey instrument was used for the pilot study with a smaller sample (N = 62). After the data from the pilot study was analyzed, additional demographic variables such as annual income, parents’ educational level, child’s services received were added to be used for the current study. Cronbach’s alpha and mean inter-item correlations revealed adequate internal consistency for the three subdomains (Table 1).

**Table 1:** Reliability analysis of the survey instrument

Subdomain	Cronbach’s alpha (α)	Mean inter-item correlations
Priorities in school readiness skills	0.827	0.370
Priorities in parent support	0.707	0.188
Challenges and barriers	0.868	0.410

The survey measured the following three domains: (1) parent demographics including cultural and linguistic characteristics (e.g., generation of immigration, English fluency, etc.) and child characteristics, (2) parents’ concepts of successful TTK (i.e., priorities in school readiness skills and parent support), and (3) challenges and barriers during the transition process.

#### Demographic Information and Child Characteristics

Demographic information of the participants included generation of immigration, years of residence in the U.S., language spoken at home, family annual income, employment status, educational level, and their relationship to the child. Responses in the parent demographics domain were measured using multiple choice questions. Moreover, child characteristic information included their current school placement, child’s current grade, child’s diagnosis other than ASD, sex, type of preschool attended, child’s main mode of communication, and services that they are currently receiving.

#### Parents’ Perspectives on Successful TTK

Parent perspectives on successful TTK were measured by the following two subdomains: (1) parents’ priorities for their child’s school readiness skills, and (2) importance of parent support in order to help their child to have a successful TTK.

In the first domain, the participants were asked, “In your opinion, how important is it to have the following skills in order to have a successful transition to kindergarten? Please rank them (1 = most important, 8 = least important). This subdomain (i.e., priorities for their child’s school readiness skill) had eight items (‘Academic readiness,’ ‘Behavioral readiness,’ ‘Social skills,’ ‘Self-help skills,’ ‘Cooperation with teachers,’ ‘Making needs known to others,’ ‘Adjusting to new environment and routine,’ ‘Separate well from parents in the morning’).

In the second domain, the participants were asked, “In your opinion, how important is it for parents to have the following skills in order to help their children to have a successful transition to kindergarten? (1 = most important, 10 = least important). This subdomain (i.e., importance of parent support) had 10 items (‘Parents’ knowledge on academic expectations, ‘Parents’ knowledge on behavioral expectations,’ ‘Building positive relationship with educators,’ ‘Having open communication with teachers when concerns arise, ‘Parents’ knowledge on U.S. school system,’ Parents’ knowledge on how to access appropriate services (e.g., speech therapy, occupational therapy, etc.),’ ‘Providing academic support at home,’ ‘Having strong relationship with other parents at school,’ ‘Being involved in school events,’ and ‘Being involved in community resources related to ASD’).

#### Challenges Experienced During TTK

In the third domain, respondents were asked, “To what extent did the following items serve as barriers in helping your child to have a successful transition to kindergarten?” There were 11 items as follows: ‘Linguistic barriers,’ ‘My lack of understanding on ASD,’ ‘My lack of knowledge about US school system,’ ‘My family’s lack of understanding on ASD,’ ‘How others may view my child’s disability/stigma,’ ‘Lack of support from my spouse,’ ‘Difficulty in building authentic relationship with educators,’ ‘Being uncomfortable discussing my child’s disability with others,’ ‘Lack of time/scheduling issues,’ ‘Difficulties in coming to an agreement in regards to placement, support, or services,’ and ‘Other life stressors.’ These items were measured using a 4-point Likert-style scale, and each point on the scale was be labeled as either “Not At All,” “Somewhat,” “Quite,” or “Very Much.” At the end of the section, participants were asked to write what other life stressors they experienced if they were not mentioned in the survey.

### Analyses

#### Demographic Information and Child Characteristics

To determine demographic information of the respondents and their child characteristics, frequency counts and percentages were calculated using SPSS version 27(IBM Corp. Released 2020).

#### Priorities for Successful Transition to Kindergarten

To answer the first research question in determining how Korean–American parents conceptualize successful transition to kindergarten, the ranking scores from the two subdomains were reverse-coded using SPSS version 27 (IBM Corp. Released 2020). The pilot study (Kim, 2019) utilized a four-point Likert-type scale for these two subdomains (i.e., Priorities in school readiness skills, Priorities in parent support) but demonstrated insufficient variances with generally high means. Moreover, the differences in mean were mostly statistically insignificant. In order to address this limitation, a decision was made to use ranking scores instead for these two subdomains in the current study.

Listwise deletion was used to handle missing data. Mean and standard deviation were calculated, and one-sample t-test was conducted to estimate 95% confidence interval for each survey item within the two subdomains.

#### Challenges and Barriers

Multiple imputation was first conducted for the variables in the Challenges and Barriers domain to address the missing data using R (R Studio Team, 2020). Missingness ranged from 0.14 to 2.36% prior to conducting missing imputation. Sources of missingness was unknown. However, a Test of Missing at Completely Random (Little, 1988) was conducted and the result suggested that the data was missing completely at random.

To answer the second research question, descriptive statistics were conducted to determine the extent to which Korean–American parents reported to experience various challenges during their children’s TTK.

#### Predictors for Challenges and Barriers

To explore possible predictors for Korean–American parents’ reported challenges during their child’s TTK, multiple regression was conducted. First, a new variable was created that reflected the sum of all the variables within the Challenges and Barriers domain. This variable was used as the DV for the model. The following variables entered the model as predictor variables in the following order hierarchically: (1) immigration generation, (2) responding parent’s primary language, (3) family’s annual income, (4) child’s mode of communication, (5) child’s school placement, (6) preschool types, (7) responding parent’s employment types, (8) co-occurring diagnosis other than ASD, (9) child’s current grade, and (10) parent’s education level. Dummy variables were created for the following predictors due to the categorical nature of the variables: immigrant generation, responding parent’s primary language, child’s mode of communication, child’s school placement, preschool types, and responding parent’s employment status. Family’s annual income was coded as follows: 1 = $15,000 or less; 2 = $15,001–$35,000; 3 = $35,001–$65,000; 4 = $65,001–$100,000; 5 = $100,000–$150,000; 6 = $150,001–$200,000; 7 = $200,001 or more. Child’s current grade entered the model in order to control for possible confound due to their varying age—Participants’ TTK experiences may be very recent for those with a kindergartener, but it may be distant for those with a 5th grader.

Assumptions for multiple regression were tested for the outcome variable and the possible predictors that would enter the model. To test the assumption of linear relationship between the IVs and the DV, scatter plots were generated between the IVs and the DV. Upon visual analysis, no violation of assumption was detected. In order to test the assumption that there is no multicollinearity in the data, analysis of collinearity statistics was conducted. VIF scores were well below 10 except for the two dummy variables (‘Preschool type: Public special education preschool’ and ‘Private preschool with English speaking staff’) with the VIF scores of 9.236 and 8.320 respectively. Further, the tolerance scores were all above 0.2, except for the aforementioned two variables with tolerances scores of 0.108 and 0.120 respectively. Since the variable ‘Preschool type: Public special education preschool’ was a significant predictor with p < 0.05 in this model, a decision was made to remove the variable ‘Private preschool with English speaking staff’ for the final model. The final model met the assumptions of multicollinearity with the VIF scores ranging from 1.084 to 1.579, and the tolerance scores ranging from 0.633 to 0.911. To test the assumption of independent residuals, the Durbin–Watson statistic was conducted. The Durbin–Watson statistic for the final model was 1.446. To test the assumption that the residuals are normally distributed, the P–P plot for the model was generated. The P–P plot for the final model suggested that the assumption of the normality has been met. Lastly, to test the assumption that there are no influential cases biasing the final model, Cook’s Distance was calculated. Cook’s Distance values were all under 1, suggesting individual cases were not unduly influencing the model. In summary, no violations to these assumptions were found in the final model. The above analyses were conducted using SPSS version 27 (IBM Corp. Released 2020).

## Results

In this section, the demographic information of the participants and their child characteristics will be reported first. Ranking information and descriptive statistics for the two domains will follow. Then protective and risk factors for the reported challenges and barriers will be presented.

### Demographic Information and Child Characteristics

Demographic information of the participants and their child characteristics are reported in Tables 2 and 3. The majority of respondents were mothers (59.9%). More than half of the respondents identified themselves to be 2nd generation immigrants (60.8%) and to have lived in the U.S. for 15 years or more, (or lived in the U.S. for their entire life) (51.9%). Further, close to half of the respondents (47.6%) reported to speak more English than Korean at home.

**Table 2:** Parent demographics (N = 212)

Variables	Frequency	%
Parents’ immigrant generation:		
1st Generation immigrants	59	27.8
1.5 Generation immigrants	24	11.3
2nd Generation immigrants	129	60.8
Relationship with child		
Mother	127	59.9
Father	83	39.2
Language spoken at home		
More Korean than English	35	16.5
Korean and English equally	74	34.9
More English than Korean	101	47.6
Years of residence in US		
0 to 5 years	5	2.4
5 to 10 years	58	27.4
10 to 15 years	37	17.5
15 years or more/born in the U.S	110	51.9
Family’s annual income		
$35,000 or less	8	3.8
$35,000–$65,000	50	23.6
$65,000–$100,000	55	25.9
$100,000–$150,000	63	29.7
$150,000–$200,000	29	13.7
$200,000 or more	6	2.8
Employment status		
Full-time	166	78.3
Part-time	19	9.0
Stay-at-home parent	24	11.3
Educational level		
Beyond bachelor’s degree	35	16.5
Bachelors’ degree	163	76.9
High school/partial post-secondary education	8	3.8
Unknown	5	2.4

**Table 3:** Child characteristics (N = 212)

Variables	Frequency	%
Sex (Boy = 1)	164	77
School placement		
Self-contained special education classroom	85	40.1
Special education classroom with some inclusion	67	31.6
General education or inclusive setting	60	28.3
Child’s current grade		
Kindergarten	58	27.4
1st Grade	31	14.6
2nd Grade	26	12.3
3rd Grade	36	17.0
4th Grade	28	13.2
5th Grade	33	15.6
Diagnoses other than ASD		
No other diagnosis	190	89.6
Has other diagnosis	14	6.6
Type of preschool attended		
Public special education preschool	128	60.4
Head start program	5	2.4
Private preschool or daycare (English Speaking)	65	30.7
Private preschool or daycare (Korean Speaking)	13	6.1
Communication modality		
Vocal communication	146	68.9
American sign language (ASL)	15	7.1
Augmented and alternative communication (AAC)	25	11.8
Pointing	21	9.9
No effective communicative modality at this time	5	2.4
Current services received (either at home or at school)		
Applied behavior analysis (ABA)	100	47.2
Speech therapy	165	77.8
Occupational therapy	82	38.7
Physical therapy	38	17.9
Other	3	1.4

Moreover, close to half of the children (40.1%) were reported to be placed in a self-contained special education classroom, and a majority of the children (89.6%) had ASD diagnosis only. More than half (60.4%) of the children started the special education program through public school from preschool. Further, a majority of the children (68.9%) were reported to be vocal communicators.

### Priorities for Successful TTK

**Fig. 1:**
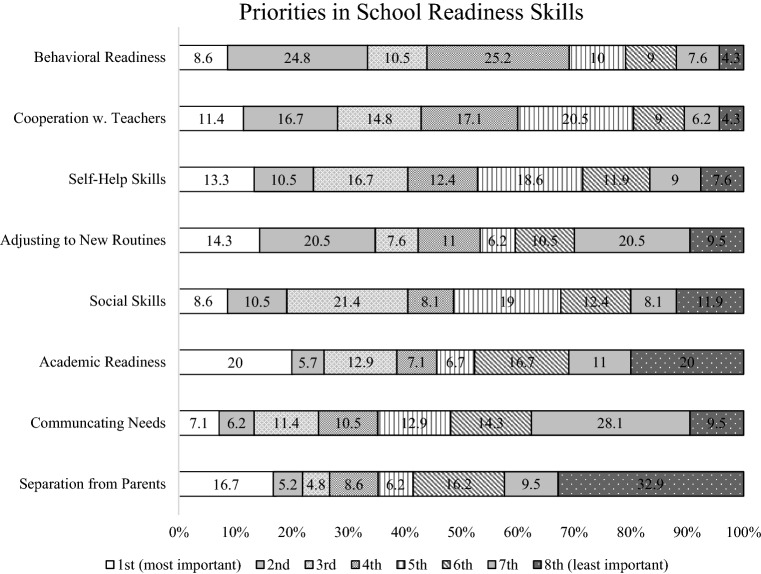
100% Stacked bar chart of each item within the subdomain: priorities in school readiness skills

Mean, standard deviation, ranking and 95% confidence intervals for each item within the first subdomain, ‘Priorities in School Readiness Skills,’ are shown in Table 4. Among the eight items within the Priorities in School Readiness Skills subdomain, ‘Behavioral readiness’ (e.g., demonstrating age-appropriate behaviors) was ranked as the highest (M = 5.17, SD = 1.92) and ‘Cooperation with teachers’ (e.g., following directions, completing tasks) was ranked as the second highest (M = 5.08. SD = 1.91). ‘Communicating needs’ (M = 3.81, SD = 2.09) and ‘Separating well with parents in the morning’ (M = 3.67, SD = 2.61) were ranked as the second lowest and the lowest, respectively. Further, percentages of parents who responded each item as first or second priority is shown in Table 5. 33.4% of the participants rated ‘Behavioral readiness’ as either the first or second priority, and 28.1% of the participants rated ‘Cooperation with teachers’ as either the first or second priority out of the eight items. Figure 1 displays the relative percentage of each rank with a 100% stacked bar for individual items within this subdomain.

**Table 4:** Descriptive statistics of the survey item in the subdomain: priorities in child’s school readiness skills (N = 210)

Rank	Priorities: child’s school readiness skills	Mean	SD	95% CI	
				Lower	Upper
1	Behavioral readiness	5.17	1.92	4.92	5.44
2	Cooperation with teachers	5.08	1.91	4.82	5.34
3	Self-help skills	4.78	2.11	4.49	5.06
4	Adjusting to new routine and environment	4.65	2.44	4.32	4.98
5	Social skills	4.52	2.13	4.23	4.81
6	Academic readiness	4.31	2.58	3.96	4.66
7	Communicating needs	3.81	2.09	3.53	4.09
8	Separate from parents	3.67	2.61	3.31	4.02

*SD* standard deviation

**Table 5:** Percentage of parents who responded each item as first or second priority (N = 210)

Rank	Priorities: child’s school readiness skills	%
1	Behavioral readiness	33.4
2	Cooperation with teachers	28.1
3	Self-help skills	23.8
4	Adjusting to new routines	34.8
5	Social skills	19.1
6	Academic readiness	25.7
7	Communicating needs	13.3
8	Separate from parents	21.9

**Fig. 2:**
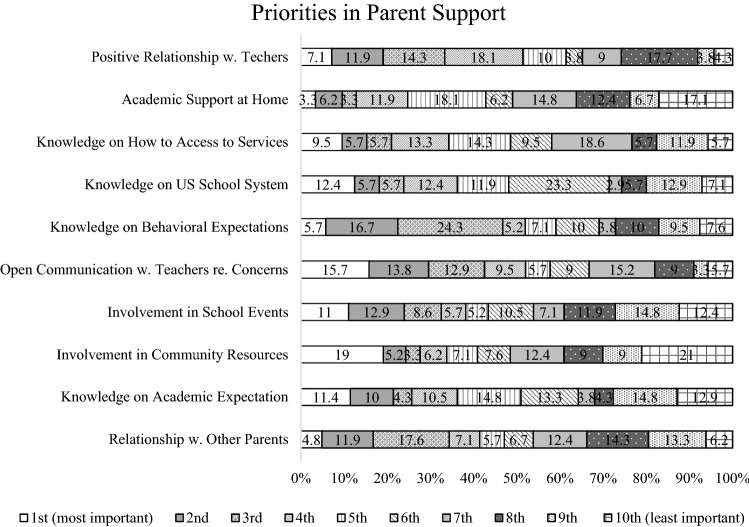
100% Stacked bar chart of each item within the subdomain: priorities in parent support

In addition, mean, standard deviation, ranking and 95% confidence intervals for each item within the second subdomain, ‘Priorities in Parent Support,’ are shown in Table 6. Among the ten items within the Parent Support subdomain, ‘Having positive relationship with teachers’ was ranked as the highest (M = 6.31, SD = 2.79) and ‘Providing academic support at home’ was ranked as the second highest (M = 6.03. SD = 2.84). ‘Knowledge on academic expectations’ (M = 5.04, SD = 3.30) and ‘Having positive relationship with other parents at school’ (M = 4.65, SD = 2.58) were ranked as the second lowest and the lowest respectively. Further, percentages of parents who responded each item as first or second priority is shown in Table 7. 19.0% of the participants rated ‘Positive relationship with teachers’ as either the first or second priority, and 9.5% of the participants rated ‘Academic support at home’ as either the first or second priority out of the ten items. Figure 2 displays the relative percentage of each rank with a 100% stacked bar for individual items within this subdomain.

**Table 6:** Descriptive statistics of survey item in the subdomain: priorities in parent support (N = 210)

Rank	Priorities: parent support	Mean	SD	CI	
				Lower	Upper
1	Positive relationship with teachers	6.31	2.79	5.93	6.69
2	Academic support at home	6.03	2.84	5.64	6.41
3	Knowledge on how to access services	6.00	2.58	5.64	6.35
4	Knowledge on US school system	5.57	2.72	5.20	5.94
5	Knowledge on behavioral expectations	5.40	2.60	5.04	5.75
6	Open communication with teachers regarding Concerns	5.39	2.79	5.00	5.77
7	Involvement in School Events	5.38	2.98	4.97	5.78
8	Involvement in community resources	5.24	3.11	4.81	5.66
9	Knowledge on academic expectations	5.04	3.30	4.59	5.49
10	Relationship with other parents	4.65	2.58	4.30	5.00

*SD* standard deviation

**Table 7:** Percentage of parents who responded each item as first or second priority (N = 210)

Rank	Priorities: parent support	%
1	Positive relationship with teachers	19
2	Academic support at home	9.5
3	Knowledge on how to access services	15.2
4	Knowledge on US school system	18.1
5	Knowledge on behavioral expectations	22.4
6	Open communication with teachers regarding Concerns	29.5
7	Involvement in school events	23.9
8	Involvement in community resources	24.2
9	Knowledge on academic expectations	21.4
10	Relationship with other parents	16.7

### Challenges and Barriers

**Fig. 3:**
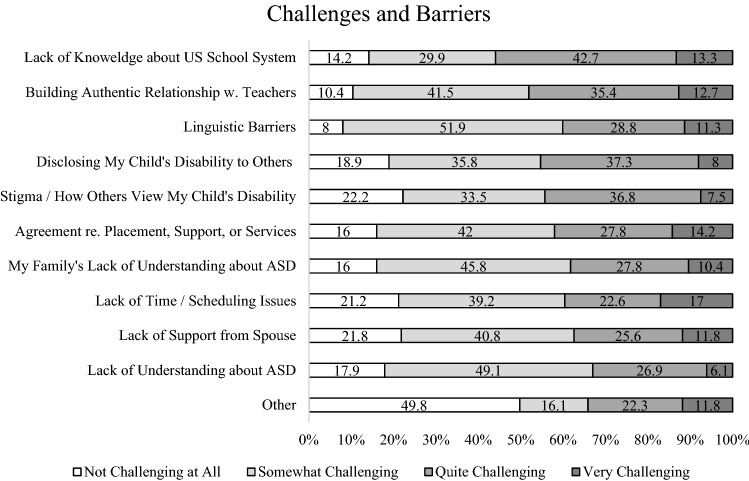
100% Stacked bar chart of each item within the challenges and barriers domain

Mean, standard deviation, ranking and 95% confidence intervals for each item within the second domain, ‘Challenges and Barriers’ are shown in Table 8. Among the ten items within the Challenges and Barriers domain, ‘Lack of knowledge about U.S. school system’ was ranked as highest (M = 2.55, SD = 0.89) and ‘Building authentic relationship with teachers’ was ranked as second highest (M = 2.51, SD = 0.85). ‘Language barriers’ (M = 2.43, SD = 0.80), ‘Disclosing child’s disability to others’ (M = 2.40, SD = 0.92), and ‘Stigma/how others may view my child’s disability’ (M = 2.35, SD = 1.00) were ranked 3rd, 4th, and 5th, respectively. Figure 3 displays the relative percentage of each rank with a 100% stacked bar for individual items within this subdomain.

**Table 8:** Ranks and descriptive statistics of survey item: challenges and barriers (N = 212)

Rank	Challenges and barriers	Mean	SD	CI	
				Lower	Upper
1	Lack of knowledge about U.S. school system	2.55	0.89	2.43	2.67
2	Building authentic relationship with teachers	2.51	0.85	2.39	2.62
3	Linguistic barriers	2.43	0.80	2.33	2.54
4	Disclosing disability to others	2.40	0.92	2.28	2.52
5	Stigma/how others view my child	2.35	1.00	2.21	2.48
6	Agreement re. placement, support, or services	2.34	0.88	2.23	2.46
7	My family’s lack of understanding on ASD	2.33	0.87	2.21	2.44
8	Lack of time/scheduling	2.30	0.90	2.18	2.42
9	Lack of spouse support	2.28	0.94	2.15	2.40
10	My lack of understanding on ASD	2.21	0.80	2.10	2.32
11	Other	1.95	1.09	1.81	2.10

*SD* standard deviation

#### Predictors for Challenges and Barriers

To explore possible predictors for challenges and barriers Korean–American parents experienced when their child on the autism spectrum transitions to kindergarten, a multiple regression analysis was conducted.

The results from the multiple regression analysis revealed a significant regression equation [F(11, 151) = 5.425, p < 0.001], with an R-square of 0.283. The sample’s predicted challenges score was equal to 29.514 + 3.039 (Immigrant generation: 1st generation = 1) + 0.895 (Parent’s primary Language: Korean = 1) − 0.904 (Family’s annual income) − 1.834 (Child’s communication modality: Vocal = 1) + 3.055 (School placement: Self-contained special education classroom = 1) + 1.590 (Preschool type: Public special education preschool = 1) − 0.901 (Preschool type: Private preschool with Korean-speaking staff = 1) − 1.325 (Parent’s employment status: stay-at-home parent = 1) − 3.27 (Co-occurring diagnosis: Yes = 1) − 0.272 (Child’s current grade) − 5.668 (Parent’s education level: College or beyond = 1). It was found that parent’s immigrant generation significantly predicted overall challenges and barriers (β = 3.039, p $\le 0.05)$ such that first generation immigrant parents reported significantly more challenges and barriers than 1.5 or 2nd generation immigrant parents. It was further found that family’s annual income significantly predicted overall challenges and barriers (β = − 0.904, p $\le 0.05)$ such that parents whose income was lower reported more challenges. Child being a vocal communicator was another significant predictor (β = − 1.834, p $\le 0.05)$ such that having a vocally communicating child was associated with lower reported challenges. Child being placed in a self-contained special education classroom significantly predicted (β = 3.055, p $\le 0.05)$ higher reported challenges. Lastly, responding parent’s educational level was found to be a significant predictor (β = − 5.668, p $\le 0.05)$ such that having a college degree or post-graduate degree predicted lower reported challenges and barriers. Parent’s primary language, preschool types, parent’s employment status, co-occurring diagnosis, or child’s current grade level were not found to be significant predictors for their overall challenges and barriers during their child’s TTK (Table 9).

**Table 9:** Multiple regression model predicting challenges and barriers experienced during TTK

Outcome	Predictors	Unstandardized beta	Standardized beta	S.E	t-ratio		p
Challenges and barriers	Immigrant generation * (1st generation = 1)	3.039	0.171	1.376	2.209		0.029*
	Parent’s primary language (Korean = 1)	0.895	0.061	1.248	0.718		0.474
	Family’s annual income *	− 0.904	− 0.316	0.220	− 4.108		0.000*
	Child’s communication modality * (Vocal = 1)	− 1.834	− 0.162	0.821	− 2.236		0.027*
	School placement * (self-contained special education classroom = 1)	3.055	0.284	0.825	3.702		0.000*
	Preschool type (public special education preschool = 1)	1.590	0.148	0.852	1.866		0.064
	Preschool type (private Korean-speaking preschool = 1)	− 0.901	0.041	− 1.630	− 0.553		0.581
	Parent’s employment status (stay-at-home parent = 1)	− 1.325	− 0.112	0.993	− 1.334		0.184
	Co-occurring diagnosis (Yes = 1)	− 3.270	− 0.134	1.803	− 1.814		0.072
	Child’s current grade	− 0.272	− 0.061	0.329	− 0.827		0.410
	Parent’s education level * (college or beyond = 1)	− 5.668	− 0.185	2.312	− 2.451		0.015*
Constant	29.514						
$R$-square	0.283						
F-ratio	5.425	p < 0.001					
n	208						

*p ≤ 0.05

## Discussion

The primary goal of the study was to examine Korean–American parents’ experiences and perspectives towards transition to kindergarten for their child on the autism spectrum. This study is exploratory and descriptive in nature that captures broad perspectives from a large sample of Korean–American parents at this unique intersectionality. Therefore, it is intended to be an important initial step to present various research agendas for future investigations to identify the perspectives that are truly unique to this specific cultural group.

The current findings align with the literature base that, although Korean parents generally consider their child’s education as highly important (Yang & McMullen, 2003), parents face various challenges and stressors during their child’s transition to kindergarten (DeCaro & Worthman, 2011; McIntyre et al., 2010; Welchon & McIntyre, 2015, 2017). Furthermore, child-level factors, family-level factors, and environmental factors that were associated with Korean–American parents’ reported challenges and barriers during their child’s TTK were examined.

### Priorities in School Readiness Skills and Parent Support

Findings from the current study regarding the priorities in school readiness skills and parent support indicate that Korean–American parents in our sample consider their children’s behavioral readiness and cooperation with teachers as the most important school readiness skills for successful TTK. Such findings are in line with the literature that, stemming from the collectivistic cultural perspectives, being a cohesive and cooperative member of the community is more valued than individualistic qualities such as communicating their own needs (Darwish & Huber, 2003). In addition, they considered building positive relationship with teachers and providing academic support at home as more important than actively involved in school events and community resources, or building relationships with other parents at the school. This finding supports the literature that, although Korean mothers are very interested in their children’s education, they are reluctant to take visible roles at school due to regarding school as an authoritative entity. Instead, they consider their role as providing the necessary support at home (Sohn & Wang, 2006; Yang & McMullen, 2003).

Moreover, both academic readiness and parents’ knowledge of academic expectations were rated as having low importance when compared to other items in this study. In contrast, McIntyre et al. (2007)‘s survey study with predominately White parents (White = 62.0%) indicated that academic expectations in kindergarten was rated as the top priority. Future studies must examine if a mismatch of cultural values between parents from dominant cultural backgrounds and Korean–American parents contribute to such difference in their perceptions on their priorities during their child’s TTK.

### Challenges and Barriers

The current study revealed that Korean–American parents in our sample rated ‘Lack of knowledge about U.S. school system’ to be the most challenging aspect of their child’s TTK. It is important to note that more than half of the sample (60.8%) was 2nd generation immigrant parents who were born and raised in the U.S. Considering their educational history in the U.S., this was a surprising finding. This implies that, in spite of their own first-hand schooling experiences in the U.S. many parents still have a difficult time navigating the school system when their child has special needs. This may be especially challenging if there were linguistic barriers and underlying cultural misalignment added to the situation. Interestingly, although 69.4% of the sample reported that they had lived in the US more than 10 years and only 16.5% of the sample reported to speak more Korean than English at home, linguistic barriers were still rated as one of the most challenging aspects. We can infer from this finding that, although the majority of these parents comfortably speak English in their home, they do encounter linguistic barriers when advocating for their child on the autism spectrum and ensuring successful TTK. It is important to note that as the present study did not include a comparison group from another cultural background, we are unable to conclude that aforementioned challenges are specific to the Korean–American population. Rather, these findings may be a reflection of challenges experienced by parents from CLD backgrounds collectively. Future studies should examine if these challenges are unique to Korean–American parents due to their cultural beliefs and misalignment.

Furthermore, as hypothesized, building an authentic relationship with teachers was rated as one of the most challenging aspects by our sample of Korean–American parents, although they rated the same item as the most important parent support they could provide. As previous literature points out building authentic relationships with teachers is one of the top priorities in successful TTK (Starr et al., 2016), yet Korean–American parents rated this as one of the most challenging factors. Literature base emphasizes the importance of family-centered practices for positive school outcomes for children (U.S. Department of Health & Human Services, 2016). Effective family-centered practices include authentic parent-educator partnerships based on mutual trust and respect, while parents are actively involved in the decision-making process. A family-centered approach involves a shift from a more child-centered focus to understanding the child in the context of the family system so educational benefits are maximized (Dunst & Espe-Sherwindt, 2016). However, Korean immigrant parents may be unaware of these components due to a cultural misalignment in the way they understand the type of relationship they should establish with educators. Therefore, in addition to reporting difficulties in building an authentic relationship with teachers, there may be a disparity in their understanding of a ‘authentic relationship.’

Moreover, there may be a complex relationship between building relationships with teachers and other challenges reported, such as stigma around the disability, being uncomfortable discussing their child’s disability with others, among others. This may be due to a mismatch in communication styles between the parents and teachers, or different cultural values towards education. Such difficulties may lead to Korean–American parents avoiding social situations or involvement at the school, which may mistakenly be seen as indifference about their child’s education. There is more to untangle around these challenges, and it is critical that future studies investigate specific challenges first-generation Korean–American parents experience while building authentic relationship with educators and navigating the school system, and examine culturally-sensitive ways for educators to engage in family-centered practices with 1st generation Korean–American immigrant parents.

Further, cultural misalignment experienced by Korean–American immigrant parents who are newer to the U.S. may have been less pronounced in this study due to the composition of the sample. Future studies should make a closer examination of the challenges experienced by Korean–American parents who are less familiar with the U.S. culture and educational systems.

### Factors that Predict or Buffer Against Challenges and Barriers

The current study revealed some factors that may predict or buffer against challenges and barriers that Korean–American parents reported during their child’s TTK. Broadly, the present study found that the child being a vocal communicator, higher family income, and higher parent educational level buffer against overall challenges and barriers that Korean–American parents reported. However, parent being a first-generation immigrant and the child being placed in a restrictive special education setting were found to predict more challenges and barriers reported by Korean–American parents.

#### Child-Level Factor: Communication Modality

Although little is known about the direct link between the child’s communication modality and their TTK process, many extant studies indicate that children’s communication skills are one of the essential school readiness skills (Eckert et al., 2008; Gill et al., 2006; LoCasale-Crouch et al., 2008; Puccioni, 2018; Welchons & McIntyre, 2017). While augmentative and alternative communication (AAC) can be supportive in facilitating communication in minimally-verbal children on the autism spectrum, evidence on the effectiveness indicates substantial individual variability (Cafiero, 2011; Schlosser & Raghavendra, 2004). Factors associated with AAC outcomes include children’s cognition, verbal imitation repertoire, and severity of ASD (Sievers et al., 2018). However, it remains unclear whether it is the communication modality itself that is directly linked to the TTK experiences, or whether the child’s underlying cognitive capacity or ineffective communication in general associated with being a non-vocal communicator is playing a role. Although challenges associated with the child’s communication modality may not be specific to Korean–American parents, it may be that having a child who is a non-vocal communicator adds to the challenges as they would need to seek additional services such as the AAC device through the school system. Such additional services could add to the burden that Korean–American parents experience. Further studies should untangle this seemingly complicated relationship between the extent of services that the child requires, possible cultural mismatch between the school system and the family, and the heightened challenges that Korean–American parents report.

#### Family-Level Factors: Immigration Generation, Family Income, and Educational Level

First-generation immigrant status was found to be associated with heightened challenges that Korean–American parents report. Moreover, higher family income and parent’s educational level were found to be protective factors for challenges and barriers reported by Korean–American parents. Recent studies (e.g., Kim et al., 2022) show that many families living in South Korea decide to immigrate to the U.S. when their child receives the diagnosis of ASD in order to access services and to provide an environment with less stigma around the disability. Based on the findings from the current study, these newly immigrated families whose members made a significant sacrifice in the hopes of providing a better life for the affected child may be at risk for higher level of challenges when the child transitions to kindergarten.

Socioeconomic status (SES) is a complex and multidimensional construct, which includes observable characteristics such as income, educational level, and occupation (Navarro-Carrillo et al., 2020). Association between SES and children’s school readiness is well-established in the literature base (e.g., Perry et al., 2018). Though we cannot confirm that such a link is heightened or specific to Korean–American families, having a low SES in addition to their complex intersectionality as an ethnic minority, immigrant, and a parent with a child with a disability may create additional barriers when their child transitions to kindergarten.

#### Environmental Factor: Restrictive School Environment

The current study revealed that the child on the autism spectrum being placed in a self-contained special education classroom as opposed to a partially- or fully-inclusive environment predicted heightened challenges that Korean–American parents report.

It is well-established in the literature that children from racially and ethnically minoritized groups and those with limited English proficiency experience added barriers in accessing autism-related services as compared to their White counterparts (Locke et al., 2017; Magaña et al., 2016; Zuckerman et al., 2014). The sample from this study reported that their lack of knowledge about the U.S. school system was the greatest challenge during their child’s TTK. Thus, navigating the special education systems during TTK is harder when the child requires a restrictive setting and more services. For instance, a child who is placed in a self-contained special education classroom may also require other intensive services at the school, which add to the burden and challenges that Korean–American parents experience. In addition to their unfamiliarity with the U.S. school system and possible linguistic barriers which can be experienced by parents from CLD backgrounds broadly, specific cultural beliefs that many Korean–American parents hold may serve as additional barriers in navigating the school system and advocating for services at school.

## Limitations

Future studies should take a closer look at the aforementioned complex relationships between reported challenges and cultural or demographic variables with special attention to this study’s limitations. First, in order to protect the confidentiality of the participants, the survey was conducted anonymously. Therefore, the participants self-reported their eligibility for the inclusion criteria. In addition, because only one parent in the family completed to the survey, we do not have information on the overall family structure. For instance, we are unsure if both parents are Korean–American, or if they have other family members (e.g., grandparents, relatives, siblings) who are actively involved in their child’s development. These factors can have an impact on the cultural influences and perceptions on their TTK experiences. Moreover, due to the exploratory nature of this study, the survey was only administered to Korean–American parents with no comparison groups. Therefore, we cannot ascertain that the findings from this study are unique to Korean–American parents with a child on the autism spectrum. Korean–American parents with a typically-developing child or parents from a different ethnic group may share similar perspectives. Therefore, future studies should expand the current research through closer examination of the perspectives that are specifically attributable to cultural variables. Further, due to the nature of brief online surveys, there is some important information that is missing from the data. For example, besides the child’s communication modality and their school placement, no data was collected where we can infer the child’s intellectual capacity or ASD severity. Similarly, besides the types of preschools they attended or current school placement, we do not have any context on the extent to which these institutions engage in family-centered practices, transition practices, or inclusive practices. As such relationships are found to be crucial for overall family quality of life (Summers et al., 2007), future studies must take a closer look at the impacts of policies and practices on the experiences of parents from minoritized ethnic groups such as Korean–Americans. Furthermore, the findings must be interpreted with caution as ranking was used to collect some of the data on the participants’ perceptions—we are unclear about the true distance between each rank. Lastly, more than half of the participants were 2nd generation Korean–American parents who were born and raised in the U.S. Therefore, some of the culture-specific findings may have been less pronounced due to their history of living in the U.S. Despite these limitations, this study provides a new insight into Korean–American parents’ lived experiences when their child on the autism spectrum transitions to kindergarten. A follow-up qualitative study about TTK experiences with Korean–American parents who are newer to the U.S. would allow for more in-depth evaluation of the culture-specific perspectives and challenges during the process.

### Practical Implications

Findings from the current study have a common underlying theme that supports the research for Korean–American parents with children on the autism spectrum, especially those who are newly immigrated as they may require more comprehensive, individualized, and culturally-responsive support during the TTK process. These parents report that they face additional layers of barriers during the transition process when a cultural or linguistic mismatch is present between parents and the school system.

Therefore, the school personnel should familiarize themselves with the underlying cultural values of families regarding the school system and ASD, and encourage families to engage in open and honest communication. For instance, educators and the school system should provide clear and culturally-responsive directions to Korean–American families about how to navigate the system and to seek support. This can be done by developing culture-specific resources to facilitate the collaboration between the educators and families. It is also important to provide these parents with opportunities to develop advocacy skills for their children with ASD in culturally-mindful ways. Finally, a parent support system for Korean–American parents created by Korean–Americans who have proficient knowledge on both the U.S. school system and Korean culture would be beneficial. This can be a parent support group where parents share their experiences and support each other to navigate the school system. This can also be Korean–American professionals (e.g., teachers, school administrators) who are familiar with the cultural variables providing support for this population.
